# Trends in Compulsory Licensing of Pharmaceuticals Since the Doha Declaration: A Database Analysis

**DOI:** 10.1371/journal.pmed.1001154

**Published:** 2012-01-10

**Authors:** Reed Beall, Randall Kuhn

**Affiliations:** 1 Josef Korbel School of International Studies, University of Denver, Denver, Colorado, United States of America; 2 Global Health Affairs Program, Josef Korbel School of International Studies, University of Denver, Denver, Colorado, United States of America; Médecins Sans Frontières, South Africa

## Abstract

**Background:**

It is now a decade since the World Trade Organization (WTO) adopted the “Declaration on the TRIPS Agreement and Public Health” at its 4th Ministerial Conference in Doha. Many anticipated that these actions would lead nations to claim compulsory licenses (CLs) for pharmaceutical products with greater regularity. A CL is the use of a patented innovation that has been licensed by a state without the permission of the patent title holder. Skeptics doubted that many CLs would occur, given political pressure against CL activity and continued health system weakness in poor countries. The subsequent decade has seen little systematic assessment of the Doha Declaration's impact.

**Methods and Findings:**

We assembled a database of all episodes in which a CL was publically entertained or announced by a WTO member state since 1995. Broad searches of CL activity were conducted using media, academic, and legal databases, yielding 34 potential CL episodes in 26 countries. Country- and product-specific searches were used to verify government participation, resulting in a final database of 24 verified CLs in 17 nations. We coded CL episodes in terms of outcome, national income, and disease group over three distinct periods of CL activity. Most CL episodes occurred between 2003 and 2005, involved drugs for HIV/AIDS, and occurred in upper-middle-income countries (UMICs). Aside from HIV/AIDS, few CL episodes involved communicable disease, and none occurred in least-developed or low-income countries.

**Conclusions:**

Given skepticism about the Doha Declaration's likely impact, we note the relatively high occurrence of CLs, yet CL activity has diminished markedly since 2006. While UMICs have high CL activity and strong incentives to use CLs compared to other countries, we note considerable countervailing pressures against CL use even in UMICs. We conclude that there is a low probability of continued CL activity. We highlight the need for further systematic evaluation of global health governance actions.

*Please see later in the article for the Editors' Summary*

## Abstract 2

Reed Beall and Randall Kuhn describe their findings from an analysis of use of compulsory licenses for pharmaceutical products by World Trade Organization members since 1995.

## Editors' Summary

**Background:**

The development of a new drug is a time-consuming and expensive process. To stimulate investment in drug development, the creators of new drugs (including the pharmaceutical companies that undertake the development and testing that is needed before any drug can be used in patients) can apply for “intellectual property rights” (a patent). Intellectual property rights protect the investments made by companies during drug development by preventing other companies from making the new drug for a fixed period of time and by providing a means by which creators of new drugs can negotiate payment from other companies for the use of their creation. Until recently, the extent and enforcement of intellectual property rights varied widely around the world. Then, in 1995, the World Trade Organization (WTO) was established. By providing a set of ground rules for trade among nations, the WTO aims to ensure that trade flows as smoothly, predictably, and freely as possible around the world. One of the founding documents of the WTO is the Agreement on Trade-Related Aspects of Intellectual Property Rights (TRIPS Agreement), which attempts to bring the protection of intellectual property rights (including patents) under common international rules.

**Why Was This Study Done?:**

Unfortunately, patent protection for drugs (pharmaceuticals) means that many medicines are too expensive for use in developing countries. While maintaining incentives for drug development, the TRIPS Agreement allows governments to license the use of patented inventions to someone else without the consent of the patent owner. Such “compulsory licensing” normally occurs only after negotiations for a voluntary license have failed, and the patent owner still receives an appropriate payment. It soon became clear that some governments were unsure of their right to use compulsory licensing and other flexibilities in the TRIPS Agreement, a situation likely to affect public health in poor countries by hindering universal access to medicines. Consequently, the WTO issued the “Declaration on the TRIPS Agreement and Public Health” at its 4th Ministerial Conference in Doha in November 2001. Reaction to the Doha Declaration, which reaffirms that the “TRIPS Agreement does not and should not prevent members from taking measures to protect public health,” has been mixed. Some experts predicted that it would increase compulsory licensing of pharmaceuticals, but others suggested that political pressure against compulsory licensing and health system weaknesses in poor countries would limit claims for compulsory licenses. In this database analysis, the researchers systematically assess the impact of the Doha Declaration on the compulsory licensing of pharmaceuticals.

**What Did the Researchers Do and Find?:**

By systematically searching media archives for reports of WTO member states considering or announcing compulsory licensing of pharmaceuticals, the researchers identified 24 verified compulsory licensing episodes in 17 nations that occurred between January 1995 and June 2011. Half of these episodes ended with an announcement of a compulsory license, and the majority ended in a price reduction for a specific pharmaceutical product for the potential issuing nation through a compulsory license, a voluntary license, or a negotiated discount. Sixteen of the compulsory licensing episodes involved drugs for HIV/AIDS, four involved drugs for other communicable diseases, and four involved drugs for non-communicable diseases such as cancer. More than half the compulsory licensing episodes occurred in upper-middle-income countries (including Brazil and Thailand). Finally, most compulsory licensing episodes occurred between 2003 and 2005. There was a smaller peak of activity in the months leading up to the Doha conference, but after 2006 activity declined substantially.

**What Do These Findings Mean?:**

Given these findings, the researchers suggest that the Doha Declaration is unlikely to have an important long-term impact on the use of compulsory licensing or on access to pharmaceuticals for communicable diseases other than HIV/AIDS in developing and low-income countries. Most notably, the researchers found no evidence of a spike in compulsory licensing episodes immediately after the Doha Declaration, and they note that the lagged spike that occurred between 2003 and 2005 could have resulted in large part from the global antiretroviral advocacy campaign. Moreover, compulsory licensing activity has diminished greatly since 2006. Thus, the researchers conclude, health advocates who pushed for the Doha Declaration reforms have had little success in engaging trade as a positive, proactive force for addressing health gaps.

**Additional Information:**

Please access these websites via the online version of this summary at http://dx.doi.org/10.1371/journal.pmed.1001154.

- The World Trade Organization provides information on intellectual property rights, on the TRIPS Agreement, on TRIPS and pharmaceutical patents, and on compulsory licensing of pharmaceuticals and TRIPS (in English, French, and Spanish); the Doha Declaration on the TRIPS Agreement and Public Health is also available
- The World Health Organization provides information on the Doha Declaration on the TRIPS Agreement and Public Health and an analysis of the implications of the Doha Declaration
- Wikipedia has pages on intellectual property rights, on the TRIPS Agreement, and on the Doha Declaration (note: Wikipedia is a free online encyclopedia that anyone can edit; available in several languages)

## Introduction

It is now a decade since the World Trade Organization (WTO) adopted the “Declaration on the TRIPS Agreement and Public Health” at its 4th Ministerial Conference in Doha. The Doha Declaration reaffirmed the right of WTO member states to apply the legal flexibility of compulsory licensing—which is a state licensing the use of a patented innovation without the permission of the patent title holder—to pharmaceutical patents under the WTO's Trade-Related Aspects of Intellectual Property (TRIPS) Agreement [1]. It also led the TRIPS Council to announce a waiver allowing states lacking strong drug production capacity to import generics under compulsory licensing [2]. Many anticipated that these actions would lead nations to claim compulsory licenses (CLs) with greater regularity, beginning with medicines for HIV/AIDS and extending into other conditions [3],[4]. Skeptics, however, doubted that many CLs would occur, given persistent political pressure against CL activity and continued health system weaknesses in poor countries [5].

### The TRIPS Agreement and Doha Declaration

The TRIPS Agreement was among the founding documents adopted by member states upon the formation of the WTO in 1995. TRIPS set out to establish a common global standard for the protection of intellectual property rights, including patents [6]. The emergence of common levels of patent protection among nations necessitated a common approach to CLs. The CL safeguard is a common feature of domestic intellectual property law and is relevant to a variety of industries [7],[8]. TRIPS Article 31 recognized the right of member states to invoke CLs, but it also sought to limit CL action by requiring a period of negotiation between the member state and the patent holder unless, as noted in subparagraph (b), it is a “case of a national emergency or other circumstances of extreme urgency.” Such negotiations create space for outcomes short of an actual CL, including negotiated voluntary licenses (VLs), price reduction, donation of the branded product, or a capitulation by the member state. Subparagraph (f) further limited CLs to the supply of the domestic market, eliminating the possibility of countries with low production capacity claiming a CL for drugs imported from another member state [9],[10].

TRIPS included transitional provisions for poor nations. Least-developed countries (LDCs), an official WTO grouping, were given a deadline for full TRIPS compliance in 2006. WTO “developing country” status is based on self-identification and includes most countries classified as low or upper-middle income by the World Bank [11]. Developing countries originally agreed to a full compliance deadline of 2000, but nations that did not previously recognize pharmaceutical product patents were given until 2005 to recognize them [12].

Article 31 became a subject of a public controversy relating to public health that peaked in 2001. At the height of the HIV/AIDS epidemic, US trade officials objected to drug licensing practices in South Africa and especially Brazil, against whom the US filed a complaint with the WTO Dispute Settlement Body (which it later withdrew) [13]. Yet, in the aftermath of the September 11 attacks in the US, the US Department of Health and Human Services publicly revisited its own CL policy in response to fears of a shortage of Bayer's ciprofloxacin (Cipro) and subsequently secured a significant discount, giving the appearance of a US double standard [14],[15]. WTO members, especially the African Group, agreed that clarification was needed, and the TRIPS Agreement's flexibilities in connection with health were added to the agenda of the WTO's 4th Ministerial Conference at Doha in November 2001 [16],[17].

Adopted on 14 November 2001, the “Declaration on the TRIPS Agreement and Public Health” opened with the following words: “We recognize the gravity of the public health problems afflicting many developing and least-developed countries, especially those resulting from HIV/AIDS, tuberculosis, malaria and other epidemics.” It went on to support “WTO members' right to protect public health and, in particular, to promote access to medicines for all” [1]. Three separate paragraphs had specific implications for the pace of CL activity from 2001 forward.

Paragraph (Par.) 5 reaffirmed Article 31, recognizing the authority of member states to grant CLs, to determine the grounds for CLs, to “determine what constitutes a national emergency,” and to define its own licensing regime without challenge. While Par. 5 did not constitute a policy change, it potentially offered a strong signal of the acceptability of pharmaceutical CLs.

Par. 6 sought to address the production capacity issue by charging the TRIPS Council with finding a “solution” for members with “insufficient or no manufacturing capabilities” to supply their domestic market. On 30 August 2003, the TRIPS Council responded with a waiver that allowed pharmaceutical-producing nations to issue a CL for the export of pharmaceutical products to nations with insufficient pharmaceutical production capabilities [2]. On 6 December 2005, the council announced its intention to make the 30 August 2003 waiver a permanent amendment [18]. The amendment has not yet been approved by the required two-thirds of member states; the deadline for ratification has been extended twice and is due to expire again at the end of 2011 [19]. Such third-party licensing became known as the Par. 6 system, and opened up the opportunity for export CLs beginning in 2003. Significant effort has been put forth to encourage member states to use the Par. 6 system to improve pharmaceutical access, including six WTO workshops and two World Health Organization (WHO)/WTO/World Intellectual Property Organization joint technical symposia [20].

Finally, Par. 7 stated an intention to extend the TRIPS compliance deadline for LDCs to 2016, a change officially put into effect by the TRIPS Council on 27 April 2002 [10]. In principle, this extension could make CLs irrelevant to LDCs. Yet in practice a great many LDCs accelerated their compliance timelines well ahead of 2016, particularly since most were preparing for a 2006 deadline. In 2004, Attaran [5] noted that 28 of the 30 the least-developed African countries had already adopted patent laws. Similarly, while the developing country transition window might have delayed CL activity until 2005, many developing countries were already compliant by 2000 [10].

### Theorized Impacts of the Doha Declaration on CL Activity

While the words of the Doha Declaration and the waiver point to the likelihood of increased CL activity, skeptics highlighted reasons to expect a more limited impact. Some emphasized barriers to the use of CLs. Many LDCs lack the production capacity, distribution networks, or buying power required to effectively use CLs [21],[22]. Par. 6 addressed production capacity but left importers dependent on willing exporters. Although Doha may have legitimized CLs for some countries, CLs could nonetheless provoke retaliatory actions including market withdrawal by the producer, informal pressure from foreign trade ministries, or formal action via the WTO [23].

Another set of skeptics argued that patents are not really the key barrier to drug access in the developing world and thus CLs were not the solution. Attaran [5] found that only 5% of items in the WHO's “Model List of Essential Drugs” [24] were developed recently enough to be eligible for patent. Even among newer drugs, many were not patented in countries with no local market to protect and little risk of generic production. Overall, a patent existed in only 1.4% of drug–country combinations. One important exception was antiretroviral drugs (ARVs), which constituted the majority of medicines that were both essential and patented [5].

The past decade has seen progress in increasing pharmaceutical access in the poorest countries, particularly with the global antiretroviral treatment scale-up, yet such improvements also relate to increased philanthropic activity, public–private partnerships, and bilateral aid. No study to our knowledge has measured the Doha Declaration's impact on the actual occurrence of CLs, and there have been relatively few systematic assessments of the Doha Declaration's impact [23],[25],[26]. While a small number of CL case registries have been constructed, these were developed for advocacy purposes and have not been subject to peer review [8],[27]–[29]. There is thus a need for a systematic analysis of CL activity.

To address this gap, we assembled a systematic database of all episodes in which a CL was either publically entertained by government officials or actually declared since the TRIPS Agreement was signed in 1995. Aside from the actual announcement of a CL, a mere threat can have a powerful effect, empowering developing nations to negotiate more aggressively with pharmaceutical houses and possibly leading to drug discounts or VLs.

## Methods

In order to capture government involvement in episodes that fell short of an actual CL, initial searches focused on media sources, rather than official government sources. We constructed a database of CL episodes meeting the following criteria: (i) the nation was a WTO member state, and (ii) there was documented evidence that the episode involved the active support of government or public officials [30]. This second criterion was essential as only nation-states have standing at the WTO and are capable of issuing a CL.

The search began with the LexisNexis media archive, which includes news items from more than 45,000 legal, news, and business sources, and industry newsletters [31]. Search criteria were broad—i.e., “(pharma! OR drug) AND (compulsory licen!)”—and generated 999 media reports of CL episodes as of 6 June 2011. Reports were reviewed manually to identify CL episodes reflecting unique country–drug combinations. This procedure was repeated using other news databases (e.g., Access World News, World News Collection, Google News), academic and legal databases (e.g., HeinOnline, ScienceDirect), and archives of relevant international organizations and lobbies (e.g., WTO, World Intellectual Property Organization, WHO, Consumer Project on Technology, Pharmaceutical Research and Manufacturers of America). These latter sources yielded no new CL episodes, but provided further background. Finally, the resulting list of CL episodes was cross-referenced against four other CL collection efforts [8],[27]–[29] as well as the designated WTO website for Par. 6 system use notifications [32]. These sources introduced no additional CL episodes.

Having compiled a list of 34 potential CL episodes in 26 countries, a subsequent verification step excluded episodes that failed to meet the public health and government involvement criteria. Searches of media, legal, academic, and industry sources were generalized to cover all mentions of the country, drug, and pharmaceutical producer together—e.g., “Ecuador Kaletra Abbott license.” Site-restricted searches were conducted on pharmaceutical firms' and government websites—e.g., “site:abbott.com compulsory licen*.” One episode involving a non-WTO member (Eritrea) was excluded. Three cases involved the negotiation of a VL between a local producer and an international patent holder, typically for export, with no government role (in South Korea, Kenya, and India). Another six involved non-government organization demands for a CL, with no evidence of government support (in the Philippines, Mexico, Colombia, Indonesia, Guinea, Cameroon, India, and Kenya).

The resulting database included 24 CL episodes in 17 nations, involving 40 pharmaceutical product patents (not including two CLs that were for all ARVs; see below). We provide a summary of each case in Text S1. For each episode we systematically identified the country, year(s), pharmaceutical product(s), and the patent holder(s). Some case studies involved a CL on multiple drugs and were difficult to consider as independent instances; therefore, we bundled these into a single episode. Ghana and Zimbabwe issued categorical CLs on all ARVs, making the exact number of affected patents difficult to determine.

CL episodes were coded according to national income, disease group, and the outcome of the episode. National income was coded as low (including World Bank low and lower-middle categories), upper-middle, or high using World Bank classifications. From the group of low-income countries (LICs), we separated out countries recognized as LDCs by WTO. We thus include four income categories: LDCs, LICs, upper-middle-income countries (UMICs), and high-income countries (HICs), Disease group was coded as HIV/AIDS, other communicable disease (CD), or non-communicable disease (NCD) based on the primary therapeutic use of the product.

Finally, four possible case study outcomes were coded: CL, VL, discount (which would include donations), or none (meaning that the CL threat was withdrawn). While these outcomes are illustrative of the diversity of CL episodes, they do not imply specific public health impacts. For example, drug donations could save more lives than a CL, but only if the number of doses donated was large. Where available, detailed data on price reductions and doses obtained are reported in Text S1, but these data typically come from unverified media sources. Health ministries, patent holders, and local producers rarely disclose such data, particularly in cases of discounts or VLs.

## Results

### General Trends and Relationships

Table 1 lists 24 unique international CL episodes from 1 January 1995 through 6 June 2011. These 24 episodes collectively involved 40 drug patents for 22 unique pharmaceutical products. Two nations applied categorical CLs on all ARVs. Years in parentheses in Table 1 indicate CL renewals, which we excluded from the overall tally. Note that Text S1 lists the references for the data in Table 1 and for the results and trends discussion contained in this section.

**Table 1:** CL episodes by year and country.

Year(s)	Nation	National Income Group	Disease	Disease Group	Total Products	Outcome
2001 (2007)	Brazil	UMIC	HIV/AIDS	HIV/AIDS	2	CL/discount
2001	Brazil	UMIC	HIV/AIDS	HIV/AIDS	1	Discount
2001	Canada	HIC	Anthrax	CD	1	Discount
2001–2003	South Africa	UMIC	HIV/AIDS	HIV/AIDS	8	VL/discount/none
2001	United States	HIC	Anthrax	CD	1	Discount
2002	Egypt	LIC	Erectile dysfunction	NCD	1	CL
2003–2004	Malaysia	UMIC	HIV/AIDS	HIV/AIDS	3	CL
2003, 2007	Brazil	UMIC	HIV/AIDS	HIV/AIDS	1	Discount
2003	Zimbabwe	LIC	HIV/AIDS	HIV/AIDS	All	CL
2004	Mozambique	LDC	HIV/AIDS	HIV/AIDS	3	CL
2004	Zambia	LDC	HIV/AIDS	HIV/AIDS	3	CL
2005–2006	Argentina	UMIC	Pandemic flu	CD	1	VL
2005–2007	Brazil	UMIC	HIV/AIDS	HIV/AIDS	1	Discount
2005–2009	Brazil	UMIC	HIV/AIDS	HIV/AIDS	1	Discount
2005	Ghana	LIC	HIV/AIDS	HIV/AIDS	All	CL
2005	Indonesia	LIC	HIV/AIDS	HIV/AIDS	2	CL
2005	Taiwan	HIC	Pandemic flu	CD	1	VL
2006–2007	India	LIC	Cancer	NCD	1	None
2006 (2010)	Thailand	UMIC	HIV/AIDS	HIV/AIDS	1	CL
2007	Rwanda	LDC	HIV/AIDS	HIV/AIDS	1	CL
2007 (2010)	Thailand	UMIC	HIV/AIDS, CVD	HIV/AIDS, NCD	2	CL
2007–2008	Thailand	UMIC	Cancer	NCD	1	Discount
2007–2008	Thailand	UMIC	Cancer	NCD	3	CL
2010	Ecuador	UMIC	HIV/AIDS	HIV/AIDS	1	CL

Totals: 24 Episodes, 17 Nations, 40 Unique Drug-Nation Combinations +2 Categorical CLs. Years in parentheses indicate CL renewals.

CVD, cardiovascular disease.

**Figure 1:**
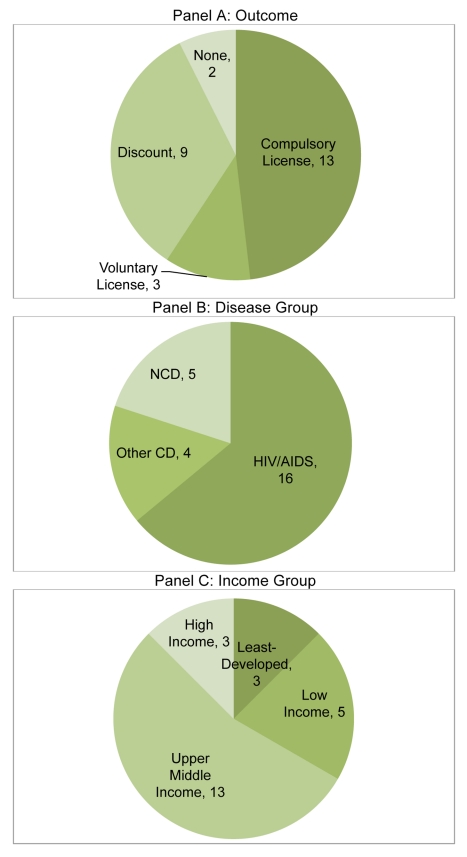
Classification of CL episodes by outcome, disease group and national income group. CL episodes classified by outcome (A), disease group (B), and national income group (C).

We next highlight key characteristics of CL episodes with respect to outcome, disease group, and national income (see Figure 1). Half of all CL episodes covered in our database ended specifically in the announcement of a CL (12 out of 24), but the great majority ended in some kind of price reduction for the potential issuing nation, whether via CL, VL, or discount. All of the findings and patterns reported below hold, whether we look at all episodes, episodes leading to any positive outcome, or only episodes in which a CL was issued.

With respect to disease group, two-thirds of CL episodes involved HIV/AIDS (16 out of 24). Of the remainder, four involved other CDs and four involved NCDs (note that one instance in Thailand involved both HIV/AIDS and an NCD). With respect to income group, a majority of CL episodes involved UMICs (13 out of 24), with three episodes occurring in HICs, five in LICs, and three in LDCs.

**Figure 2:**
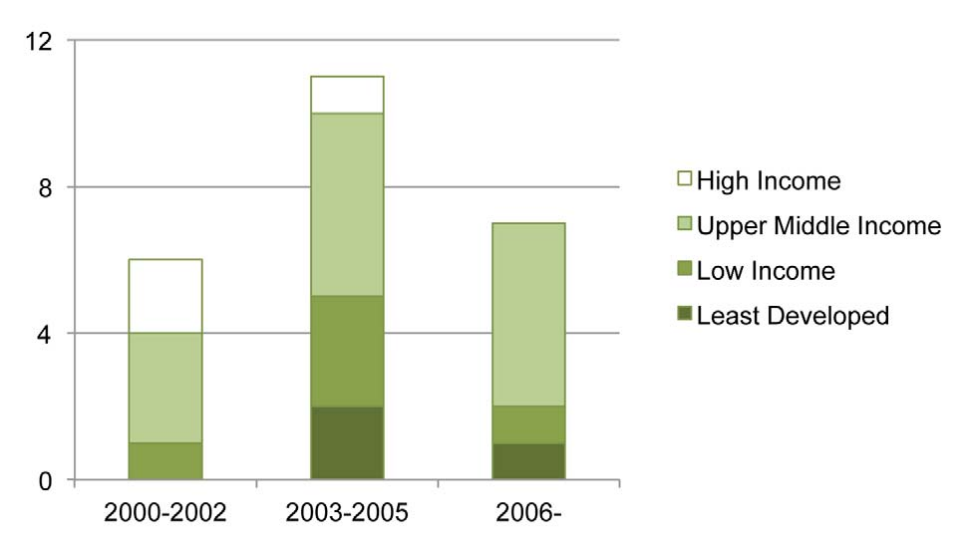
Classification of CL episodes by time period and national income group.

To better understand the trends visible in Table 1 and the development of CL activity over time, we next classified the episodes into three distinct periods of activity. We found no evidence of CL activity between 1995 and 2000 (see Figure 2). The first period of CL activity took place in the months before the 2001 Doha conference. This period included five CL episodes involving three UMICs and two HICs. South Africa and Brazil both made widely publicized policy changes and CL threats that resulted in significant drug discounts and VLs. After the anthrax attacks in the US in 2001, Canada issued a CL aimed at stockpiling medicines. The US publicly reexamined its own CL policy and subsequently obtained discounts on ciprofloxacin (Cipro) from Bayer. No nation invoked a CL for NCD drugs during this time frame. The period immediately following Doha saw only one CL. Egypt, an LIC, issued a CL for the male erectile dysfunction drug sildenafil (Viagra).

The period from 2003 to 2005 saw the greatest volume of CL activity, especially for ARVs. Nearly half of all of the documented CL episodes occurred in this period (11 of 24). ARVs accounted for nine of the 11 episodes, including two categorical CLs on all HIV/AIDS medicines. Although this spike in CL episodes coincided with the 30 August 2003 export waiver decision, none of these episodes were export CLs, and many of these episodes preceded August 30. Taiwan and Argentina entertained CLs for avian influenza medicines, receiving VLs in both cases. This period also included the greatest variety of countries, including two LDCs, three LICs, five UMICs, and one HIC.

The period from 2006 to June 2011 saw a substantial decline in activity. There have been seven CLs entertained by four countries, with six of the seven episodes involving UMICs. The current era is dominated by the activities of Thailand. Thailand began with HIV/AIDS but quickly became the most active issuer of CLs for NCD drugs, including drugs for cardiovascular diseases and cancer. India entertained a CL for the cancer drug imatinib mesylate (Gleevec) in 2006, but didn't issue a CL. Two other nations issued CLs for ARVs. In 2007, Rwanda became the only LIC to claim a CL for an imported drug when it bought lamivudine/nevirapine/zidovudine (Apo-TriAvir) from Apotex, a Canadian firm. This well-studied episode illustrated many pitfalls in the existing Par. 6 system, including a variety of costly bureaucratic delays and a price that was still higher than for comparable Indian generics [33]–[35]. The current period has seen no CLs relating to CDs other than HIV/AIDS.

## Discussion

### Scrutinizing CL Activity in Low and Middle-Income Countries

While the Doha Declaration and the ensuing waiver process had many motivations and intended effects, those mentioned most prominently in the actual text related to improving access to drugs in poor countries, particularly for CD [1]–[3]. It is therefore striking and perhaps controversial that we observe a higher level of CL activity in UMICs than in LICs and LDCs. While the goal of this study was not to compare rates or propensities to carry out CLs across country groups, it is nonetheless worth considering whether middle-income countries (MICs) are in fact more likely to carry out CLs, and if so, why? We address some potential explanations. Perhaps the infrequency of CLs by LDCs is an artifact of the transition process, specifically Par. 7, which delayed the TRIPS transition deadline for LDCs to 2016? If LDCs were not even honoring patents through the study period, then we have clearly not established an apples-to-apples comparison. Yet as we noted above, most LDC members of WTO had actually implemented TRIPS-compliant patent laws by 2004. Two LDCs, Mozambique and Zambia, did in fact claim CLs in 2004, suggesting the CLs were indeed a possibility in LDCs.

Perhaps a more compelling possibility is that LDCs simply had alternative mechanisms for gaining access to drugs that were not available to other countries. This is most relevant in the case of the global ARV scale-up, in which Indian generics producers worked closely with the Clinton Foundation and other non-government organizations to export generic HIV/AIDS drugs developed prior to 2005 (and thus not subject to patent in India) to HIV-affected nations [36],[37]. While India retained the right to export non-patented antiretroviral treatments, by the end of the developing country transition period, only LDCs whose patent transition windows had been extended to 2016 would have been allowed to import such medicines. This again could cast some doubt on the comparability of the CL environment across country income categories. We note, however, that this mechanism would only affect our results with respect to HIV/AIDS, the disease which actually saw the greatest CL activity. We found no evidence of this export practice for drugs related to any other diseases.

We thus argue that there was indeed a low propensity to carry out CLs among LDCs and LICs, and that our finding is not a mere artifact of Doha Declaration transition periods. Our data cannot, however, distinguish between a low rate of CL activity for drugs that were patented or simply a low propensity for many products to be patented in the first place, as was found in research based on the pre–Doha Declaration period [5]. Yet either explanation would support a similar interpretation, that CLs have little direct relevance to poor countries. Producers send a powerful signal of the threat of generic production or distribution of drugs in a country when they choose not to patent a drug at all. Future studies could assess whether the Doha Declaration has had any impact on subsequent patenting behaviors of pharmaceutical companies.

Most CL activity, including all activity since mid-2007, took place in UMICs, a phenomenon we interpret in terms of economic, epidemiologic, and health system transitions. Relative to LICs, UMICs have the production and distribution capacity to carry out CLs. UMICs may also have the weight within the political economic system to withstand political pressure and threats of retaliatory action. Demand for CLs is further explained by the epidemiologic transition, as the shift from CDs to NCDs being the leading cause of morbidity induces a rise in pharmaceutical unit costs and treatment durations [38]. Whereas HICs have well-developed systems for discovery of advanced medicines and reimbursing patients for the use of costly medicines, these systems may be nascent in many UMICs [39].

The impetus for CL activity may be particularly high in UMICs facing a double burden of disease involving HIV/AIDS. First, HIV/AIDS places additional financial burdens on health systems that create a need to cut costs elsewhere. Second, HIV/AIDS familiarizes health systems with the costs and burdens of long-term palliative care. Finally, HIV/AIDS patients themselves can experience an extraordinarily high rate of NCD. This could explain the progression that was seen in CLs issued by Thailand: HIV/AIDS CLs in 2006, CLs for the blood thinner clopidogrel bisulfate (Plavix) and the AIDS medication lopinavir/ritonavir (Kaletra) in January 2007, followed by CLs for cancer medicines in 2007 and 2008.

### Conclusion

Almost ten years after the Doha Declaration, we examined the subsequent occurrence of CL episodes, an important direct indicator of treaty impact. We found evidence of a modest number of pharmaceutical CL episodes, especially when considering threatened CLs as well as CLs actually issued. Most episodes were related to the drugs for HIV/AIDS. These episodes occurred not immediately after the Doha Declaration, but in a period between 2003 and 2005. It is beyond the scope of this work to ascertain whether this lagged spike resulted from the Doha Declaration itself, from the global ARV advocacy campaign, or from a combination of the two. Either way, the occurrence of so many CLs, even among countries that were technically exempted from full TRIPS compliance until 2016, suggests that CLs had achieved increased legitimacy as a health action, particularly given that no CLs were declared before 2001. Yet just as notable is the marked decrease in CL activity from 2006 forward. Future research should validate this apparent decline and assess possible explanations.

While CLs are available to all nations, the rhetoric preceding the Doha Declaration and the language of the declaration itself emphasized the need to increase pharmaceutical access in poor countries facing high rates of CD. We found few instances of CLs for CDs other than HIV/AIDS, and these few related to potential pandemic diseases (influenza, anthrax) in rich countries. We found no CLs for high-impact diseases with patented treatments such as malaria, multi-drug-resistant tuberculosis, or sepsis. After the Par. 6 export waiver policy, we observed just one export CL, a case involving Canada and Rwanda that demonstrated many of the obstacles inherent in the export waiver system. We thus conclude that the barriers to CL use in LDCs and LICs go well beyond the lack of production capacity, and likely extend to health system incapacity, political pressure against CLs, and the legislative difficulties of issuing a CL.

Instead, a majority of CL activity occurred within UMICs, including a number of episodes involving drugs for NCDs. Given the opportunities and incentives for UMICs to employ CLs, we may ask why MICs have not rushed to issue CLs on a far wider range of medicines. In fact, MICs face considerable pressure from internal industrial lobbies and foreign governments to honor intellectual property rights [23]. Patent recognition may also be seen as an opportunity to attract foreign investment and technology transfer. Many regional or bilateral trade agreements now include so-called TRIPS-plus provisions that expand patent rights or limit generic production capacity [23]. While UMICs may be the group most likely to employ CLs, even they may be quite unlikely to do so under existing rules.

Given these considerations, we argue that there is a high probability that the efforts put forth during the Doha conference in regard to pharmaceutical CLs will have a negligible long-term impact on the regular use of CLs or on global access to pharmaceuticals. This may be unsurprising given that TRIPS is neither a health nor a pharmaceutical access framework, but a trade framework. Nevertheless, health advocates who pushed for the Doha Declaration reforms have had far less success in engaging trade as a positive, proactive force for addressing health gaps.

In closing, we note that while systematic impact evaluations have gained wide acceptance in population health research, they can be equally valuable at the global scale. There is a great need for systematic evaluation of existing global health agreements, treaties, and conventions. Such research could ultimately enhance the impact of global health governance.

## Supporting Information

Text S1 CL case study summaries.
(DOCX)

## Abbreviations

ARV: antiretroviral drug
CD: communicable disease
CL: compulsory license
HIC: high-income country
LDC: least-developed country
LIC: low-income country
MIC: middle-income country
NCD: non-communicable disease
Par.: Paragraph
TRIPS: Trade-Related Aspects of Intellectual Property
UMIC: upper-middle-income country
VL: voluntary license
WHO: World Health Organization
WTO: World Trade Organization
