# Supplementary material for: Trends in Compulsory Licensing of Pharmaceuticals Since the Doha Declaration: A Database Analysis
Source: PLoS Med. 2012 Jan 10;9(1):e1001154. doi: 10.1371/journal.pmed.1001154 (PMC3254665; doi:10.1371/journal.pmed.1001154)
Supplement: Text S1 — CL case study summaries. (DOCX) [file pmed.1001154.s001.docx]

Text S1. CL Case Study Summaries

Table of Contents

Argentina 1

Brazil (and Bristol Myers Squibb) 2

Brazil (and Merck) 3

Brazil (and Roche) 4

Brazil (and Abbott) 5

Brazil (and Gilead) 6

Canada 7

Ecuador 8

Egypt 9

Ghana 10

India 11

Indonesia 12

Malaysia 13

Mozambique 14

Rwanda 15

South Africa 16

Taiwan 17

Thailand (and Merck) 18

Thailand (and Abbott, Sanofi-Aventis) 19

Thailand (and Novartis) 20

Thailand (and Novartis, Sanofi-Aventis, Roche) 21

United States 22

Zambia 23

Zimbabwe 24

# Argentina

**Year(s):** 2005-2006

**National Income:** Upper Middle

**Pharmaceutical Firm(s):** Roche

**Drug(s):**  Oseltamivir (Tamiflu)

**Disease:** Avian flu/Pandemic Flu

**Disease Scenario Type:** CD

**Outcome(s):** VL

**Case Study Summary:** In 2005, Argentina threated to issue a compulsory license for Tamiflu. Burke reports: “Health Minister Gines Gonzalez Garcia told Reuters. ‘We don't have to wait until the virus appears to say we are going to do this and produce the drug. We must have it in case the virus appears.’…Argentina said it would exercise its right, enshrined in international accords, to issue a compulsory license to produce generic copies of patented drugs in a medical emergency.” Pharma and Healthcare writes in April 2006: “Local press reports claim that Swiss drugmaker is to concede voluntary licences for its supposed avian flu treatment Tamiflu (oseltamivir) in Argentina. According to a company statement, there is 'no conflict' over the patent for the drug, and will create a 'co-operative' production network for delivery under a new 5mn dose government order for the drug. Two local firms are now reported to have initiated production of Tamiflu. In January, the company also invested US$2.4mn in a new quality control laboratory in Buenos Aires.” Nevertheless, Bucknell and Love report in 2007 that the Roche’s patent on Tamiflu was later invalidated in Argentina.

**Selective Bibliography:**

Bucknell D (22 October 2007) The global IP scorecard for pharma and biotech compulsory licenses. Mondaq Business Briefing. Available: <http://www.mondaq.com/australia/article.asp?articleid=53442>. Accessed 5 June 2011.

Burke H (19 October 2005) Argentina says plans to produce own bird flu drug. Reuters.

Available: <http://www.twnside.org.sg/title2/health.info/twninfohealth012.htm>. Accessed 11 June 2011.

Pharma and Healthcare (April 2006) Roche to voluntarily licence Tamiflu in Argentina.

Company Finance Alert. Available: <http://www.pharmaceuticalsinsight.com/file/33495/roche-to-voluntarily-licence-tamiflu-in-argentina.html>. Accessed 11 June 2011.

Love JP (6 May 2007) Recent examples of the use of compulsory licenses on patents.

Knowledge Ecology International. Available: <http://www.keionline.org/misc-docs/recent_cls.pdf>. Accessed: 6 June 2011.

# Brazil (and Bristol Myers Squibb)

**Year(s):** 2003, 2007

**National Income:** Upper Middle

**Pharmaceutical Firm(s):** Bristol Myers Squibb

**Drug(s):**  Atazanavir sulfate (Reyataz)

**Disease:** HIV/AIDS

**Disease Scenario Type:** HIV/AIDS

**Outcome(s):** Discount

**Case Study Summary:** Sturm writes: “Following lengthy negotiations, the Brazilian government has reached agreement with US drug-maker Bristol-Myers Squibb (BMS) to purchase its new protease inhibitor Reyataz (atazanavir sulfate) at a hugely discounted price. The agreement makes Reyataz, a drug used in combination with other anti-retroviral drugs (ARVs) for the treatment of HIV/AIDS, available to the Brazilian government at a unit price of US$3.25, a 76.4% discount on the current market price of US$13.80. Brazil, famous for its successful drug price negotiations, said this latest discount was the largest it had ever obtained from a multinational drug-maker. The Ministry of Health estimates that the discount will translate into a yearly saving of R$191m (US$65m). Brazil spends over R$500m (US$170.2m) a year on procuring AIDS drugs...the deal with BMS increases the pressure on the other three pharmaceutical companies to follow suit, while the threat of compulsory licenses made earlier by the Brazilian Ministry of Health has also upped the stakes.” Shankland reports: “under a deal agreed in January 2007, Bristol-Myers Squibb (BMS) announced that it had saved the health ministry more than US$70 million by cutting the price of the per-patient daily dose of Reyataz (atanazavir) from US$6.58 to US$6.13. BMS stated at the time that this deal had preserved it from the threat of compulsory licensing.”

**Selective Bibliography:**

Sturm, T. (2003). Brazil Negotiates Substantial ARV Discount from BMS. World Markets Research Centre.

Shankland B (26 February 2007) Judge in Brazil Eases Path to Compulsory Licence for Efavirenz. Global Insight. Available: LexisNexis

Tamayo M (November 28, 2007) Debate Rages Over Patent Laws in Brazil. Global Insight. Available: LexisNexis

# Brazil (and Merck)

**Year(s):** 2001 (2007)

**National Income:** Upper Middle

**Pharmaceutical Firm(s):** Merck

**Drug(s):**  Efavirenz (Stocrin or Sustiva) and Indinavir (Crixivan)

**Disease:** HIV/AIDS

**Disease Scenario Type:** HIV/AIDS

**Outcome(s):** Discount/CL

**Case Study Summary:** In 1997, Brazil began a highly praised anti-AIDS program that provides free treatment. After the program’s inception, Brazil’s first CL threat targeted Merck in 2001. Brazil initially rescinded the threat after Merck afforded discounts. Rich writes: “Patent-breaking threats by the Brazilian government last March prompted Merck & Company to reduce the price of two AIDS drugs, indinavir and efavirenz, by around 60 percent in Brazil.” Petersen and Rother write: “Government officials estimated that the price reductions on Merck's two drugs would save the Health Ministry about $40 million a year. Brazil's annual drug budget for its estimated 550,000 H.I.V.-positive patients is $305 million, with Stocrin alone accounting for more than 10 percent of that total.” Still, the annual cost to Brazil was $42 million. Renegotiations have happened intermittently. Merck had offered to reduce the price of Efavirenz from $1.59/pill to $1.10, but in 2007, Brazil issued a CL in order to buy from an Indian generic drug producer at $0.45/pill.

**Selective Bibliography:**

Merck & Co (4 May 2007) Statement on Brazilian Government's Decision To Issue Compulsory License for STOCRIN. Available: http://www.merck.com/newsroom/news-release-archive/corporate/2007_0504.html. Accessed 5 June 2011.

Petersen M, Rohter L (31 March 2011) Maker agrees to cut price of 2 AIDS drugs in Brazil. Available: <http://www.nytimes.com/2001/03/31/world/maker-agrees-to-cut-price-of-2-aids-drugs-in-brazil.html>. Accessed 5 June 2011.

Rich JL (1 September 2001) Roche reaches accord on drug with Brazil. Available: <http://www.nytimes.com/2001/09/01/business/roche-reaches-accord-on-drug-with-brazil.html>. Accessed 5 June 2011.

Sequera V (4 May 2007) Brazil bypasses patent on Merck's AIDS drug. BBC. Available: <http://news.bbc.co.uk/2/hi/americas/6626073.stm>. Accessed 5 June 2011.

Sherman P, Oakley EF (2004) Pandemics and panaceas: The World Trade Organization's efforts to balance pharmaceutical patents and access to AIDS drugs. Am Bus LJ 41(Winter/Spring): 353-411

# Brazil (and Roche)

**Year(s):** 2001

**National Income:** Upper Middle

**Pharmaceutical Firm(s):** Roche

**Drug(s):** Nelfinar (Viracept)

**Disease:** HIV/AIDS

**Disease Scenario Type:** HIV/AIDS

**Outcome(s):** Discount

**Case Study Summary:** Sherman and Oakley write: **“**Roche Holding…finally capitulated to Brazilian pressure to lower prices or face a compulsory license. On August 22, 2001, after the failure of lengthy price negotiations between Brazil and Roche, Brazil threatened to issue a compulsory license on Roche's anti-AIDS drug, Viracept, thus breaking the patent held by Roche on Viracept in Brazil. Health Minister Jose Serra stated he had started the process to issue such a license to allow Far-Manguinhos to produce Viracept pursuant to a provision of Brazilian law that allows such a license in the event of abusive prices. The Minister stated that Brazil bought 82 million units of Viracept each year for $88 million, or 28 percent of the entire government spending for a year on its AIDS program. The government estimated domestic production would save it 40 percent, or $35 million a year. Roche spokespersons expressed surprise at the announcement, noting that Brazilian prices were already 50 percent of the cost in the US, the current negotiations concerned Brazil's needs for Viracept in 2002, and Roche had plans to begin producing Viracept in Brazil in 2002. In fact, Roche's current contract with Brazil did not even expire until 2002. Nevertheless…in August 31, 2001, the company announced that it had reached agreement with Brazil to cut prices on Viracept by an additional 40 percent, to a final price which is 30 percent of the US' cost.”

**Selective Bibliography:**

Rich JL (1 September 2001) Roche reaches accord on drug with Brazil. Available: <http://www.nytimes.com/2001/09/01/business/roche-reaches-accord-on-drug-with-brazil.html>. Accessed 5 June 2011.

Reuters (23 August 2001) Brazil plans to ignore patent on AIDS Drug. Available from LexisNexis.

Sherman P, Oakley EF (2004) Pandemics and panaceas: The World Trade Organization's efforts to balance pharmaceutical patents and access to AIDS drugs. Am Bus LJ 41(Winter/Spring): 353-411

Zolotaryova V (2008) Are we there yet? Taking TRIPS to Brazil and expanding access to HIV/AIDS medication. Brook J Int’l L 33:1099-1126

# Brazil (and Abbott)

**Year(s):** 2005-2007

**National Income:**  Upper Middle

**Pharmaceutical Firm(s):** Abbott

**Drug(s):**  Lopinavir+ritonavir (Kaletra)

**Disease:** HIV/AIDS

**Disease Scenario Type:** HIV/AIDS

**Outcome(s):** Discount

**Case Study Summary:** Bjornberg writes: “In June 2005, Brazil's Health Ministry threatened to infringe the patent on Kaletra, an anti-AIDS medication owned and developed by Abbott Laboratories; Brazil said that it would produce a generic version of the drug in government laboratories unless Abbott agreed to lower the price or voluntarily grant patent rights to the Brazilian government...in justifying its position, the health ministry asserted that the price of Kaletra previous to the agreement was so high that it endangered the sustainability of Brazil's AIDS program.” Bjornberg notes that the Brazilian government estimated ARV costs to be $4,137 per patient per year. Reuters writes: “Brazil's Health Minister Humberto Costa said…that his ministry was issuing a compulsory license order for the Kaletra antiretroviral (ARV) drug on the Sao Paulo office of…Abbott Laboratories Inc... Under the compulsory license… the price would be slashed from $1.17 a pill to 68 cents.” Negotiations continued. Abbott reported reaching an agreement with Brazil in 2007 at a “preferential rate of $1,000 per patient per year.”

**Selective Bibliography:**

Abbott (2008) 2008 Global Citizenship Report. Available: <http://www.abbott.com/static/cms_workspace/content/document/Citizenship/Reports/gc_report_2008.pdf>. Accessed on 5 June 2011.

Bjornberg J (2006-2007) Brazil's recent threat on Abbott's patent: resolution or retaliation? Nw J Int’l L & Bus 27: 199-226.

Griffiths K (9 July 2005) Abbott set to strike deal with Brazil to cut cost of AIDS drug. The Independent. Available: <http://www.independent.co.uk/news/business/news/abbott-set-to-strike-deal-with-brazil-to-cut-cost-of-aids-drug-498052.html>. Accessed 5 June 2011.

Reuters (6 July 2005) Drugs body says Brazil to lose from AIDS patent move. Available on LexisNexis.

Sequera V (4 May 2007) Brazil bypasses patent on Merck's AIDS drug. Associated press financial wire. Available from LexisNexis.

# Brazil (and Gilead)

**Year(s):** 2005-2009

**National Income:** Upper Middle

**Pharmaceutical Firm(s):** Gilead

**Drug(s):** Tenofovir (Viread)

**Disease:** HIV/AIDS

**Disease Scenario Type:** HIV/AIDS

**Outcome(s):** Discount

**Case Study Summary:** Reuters writes in 2005: “Brazil requested…Gilead Sciences Inc. to grant ‘voluntary licensing’ of drug technology so it can keep its much-copied AIDS program afloat…‘We expect to cut by half what we currently pay,’ the ministry's health control secretary, Jarbas Barbosa, said.” Gilead reports that in May 2006, the firm afforded Brazil a 50 percent discount. Reuters reports in 2008: “This year, 31,300 people in Brazil are expected to be treated with tenofovir at a cost of $1,387 per person. The annual cost per person for the 180,000 people included in Brazil's HIV/AIDS program is about $2,500 worth of medicines each year.” The Health Ministry declared tenofovir to be a drug of public interest in 2008. The tenofovir patent was denied in the same year. Preparations began for generic production of the drug in 2009.

**Selective Bibliography:**

Brazilian Ministry of Health (16 December 2010) Clipping – 16 de dezembro de 2010 – quinta-feira. Available: <http://portal.saude.gov.br/portal/arquivos/pdf/clipping_16_12_2010.pdf>. Accessed 5 June 2011.

Gilead (1 March 2010) Form 10-K: annual report which provides a comprehensive overview of the company for the past year. Available: <http://www.gilead.com/pdf/Gilead10K2009.pdf>. Accessed 5 June 2011.

Gilead (27 February 2007) Form 10-K: annual report which provides a comprehensive overview of the company for the past year. Available <http://www.gilead.com/pdf/GILEADSCIENCESI10K.pdf>. Accessed 5 June 2011.

Palermo MP (10 April 2008) Brazil may reject Gilead's AIDS drug patent. Available: <http://www.reuters.com/article/2008/04/10/brazil-aids-gilead-idUSN1034936120080410/>. Accessed 5 June 2011.

Reuters (16 March 2005) Brazil takes step toward breaking AIDS patents. Available: <http://www.globalhealth.org/news/article/5752>. Accessed 5 June 2011.

Sequera V (29 Jun 2005) Brazil expects U.S. AIDS drug makers to comply with demands. Available: LexisNexis.

# Canada

**Year(s):** 2001

**National Income:** High

**Pharmaceutical Firm(s):** Bayer

**Drug(s):** Ciproflaxin (Cipro)

**Disease:** Anthrax

**Disease Scenario Type:** CD

**Outcome(s):** Discount

**Case Study Summary:** Canada issued a CL as a precaution during the bioterrorist attacks in September and October of 2001 and began to organize the generic production. After a CL was announced, price negotiations between Canada and Bayer commenced. The parties eventually reached an agreement on a discounted price of $2.50 cents per pill, and Canada rescinded compulsory licensing activities on Cipro, given the discount. After the United States negotiated a price of $0.95 per pill for 1 million doses (a quantity much larger than the Canadians originally sought), Canadian officials were able to secure the same price as the United States.

**Selective Bibliography:**

Bayer Corporation (24 June 2002) Form 20-F. United States Securities and Exchange Commission. Available: [www.investor.bayer.com/user_upload/469/](http://www.investor.bayer.com/user_upload/469/). Accessed 5 June 2011.

Ferrone JD (2003) Compulsory licensing during public health crises: bioterriorisms mark on global pharmaceutical patent protection. Suffolk Transnatl Law R 26: 385-410.

Harmon A, Pear R (19 Oct 2001) A nation challenged: the treatment; Canada overrides patent for Cipro to treat anthrax. New York Times. Available: <http://www.nytimes.com/2001/10/19/business/nation-challenged-treatment-canada-overrides-patent-for-cipro-treat-anthrax.html>. Accessed 5 June 2011.

Parliament of Canada (25 October 2001). Debates of the Senate (Hansard), 1^st^ Session, 37^th^ Parliament, Volume 139, Issue 63. Available: <http://www.parl.gc.ca/Content/Sen/Chamber/371/Debates/063db_2001-10-25-e.htm>. Accessed 5 June 2011.

# Ecuador

**Year(s):** 2010

**National Income:**  Lower Middle

**Pharmaceutical Firm:** Abbott

**Drug:** Lopinavir + ritonavir (Kaletra)

**Disease:** HIV/AIDS

**Disease Type Scenario:** HIV/AIDS

**Outcome:**  CL

**Case Study Summary:** Saez writes: “Ecuador granted its first compulsory license for a patented pharmaceutical since declaring last year that it would utilize international rules allowing it to do so. The compulsory license was granted for ritonavir, an antiretroviral drug, on 14 April to Eskegroup SA, the local distributor for Cipla, an Indian generic pharmaceutical producer, according to Andrés Ycaza Mantilla, head of the Ecuadorean intellectual property office (IEPI). The owner of the patent is Abbott Laboratories, a US pharmaceutical manufacturer. Eskegroup will pay royalties to Abbott for using the license under the term of the compulsory license. The compulsory license has been granted for the time that was left on the patent, until 30 November 2014.” The Targeted News Service writes: “Ecuador's compulsory license immediately reduced the cost of a major public HIV drug purchase last week by 27 percent.”

**Selective Bibliography:**

Bloeman S (26 April 2010) Compulsory licenses in Ecuador will lower the price of medicines: countries in the region should follow Ecuador’s example. Health Action International (HAI). Available: [www.conasa.gov.ec/codigo/boletin/medica/030510(4.18).doc](http://www.conasa.gov.ec/codigo/boletin/medica/030510(4.18).doc). Accessed 5 June 2011.

Claux D (7 May 2010) Kaletra's compulsory license divides opinion in Ecuador. World Markets Research Center. Available: LexisNexis.

Ministry of Public Health, Ecuador (26 April 2010) Correa sobre Medicamentos. Available: [www.conasa.gov.ec/codigo/boletin/medica/260410.doc](http://www.conasa.gov.ec/codigo/boletin/medica/260410.doc). Accessed 5 June 2011.

Saez C (22 April 2010) Ecuador grants first compulsory license, for HIV/AIDS drug. Intellectual Property Watch. Available: <http://www.ip-watch.org/weblog/2010/04/22/ecuador-grants-first-compulsory-licence-for-hivaids-drug/>. Accessed 5 June 2011.

Valdiviseso J (28 October 2009) Ecuador to make cheap versions of patented drugs.

Associated Press Financial Wire. Available: LexisNexis.

# Egypt

**Year(s):** 2002

**National Income:** Lower Middle

**Pharmaceutical Firm(s):**  Pfizer

**Drug(s):** Sildenafil (Viagra)

**Disease:** NCD

**Disease Scenario Type:** NCD

**Outcome(s):** CL

**Case Study Summary:** Bird and Cahoy write: “In 2002, after four years of effort, Pfizer finally received regulatory approval to enter the Egyptian market with their popular drug Viagra. Local well-connected drug manufacturers responded by pressuring the Egyptian Ministry of Health, accusing the Ministry of helping MNEs exploit Egypt's poor. Only two months after Pfizer's entry into the Egyptian market, the Ministry decided to grant similar authorization to produce Viagra to all Egyptian companies who applied to do so. The Ministry of Health cited the interests of the poor and the fact that the 2005 deadline for developing countries to comply with TRIPS had not yet expired. Pfizer was, naturally, ‘furious’ at the decision. The decision led Pfizer to ‘slam the brakes’ on a state-of-the-art production facility in Egypt.”

**Selective Bibliography:**

Bird R, Cahoy D (Summer 2008) The impact of compulsory licensing on foreign direct investment: a collective bargaining approach. Am. Bus. L.J. 45(2): 283-330

Fair R (Winter 2009) Does Climate Change Justify Compulsory Licensing of Green Technology? BYU Int'l L. & Mgmt. Rev. 6: 21-41

Feldman J (Spring 2009) Compulsory licenses: the dangers behind the current practice. J. Int'l Bus. & L. 8:137-167

McGill A (Fall 2009) Compulsory licensing of patented pharmaceuticals: why a WTO administrative body should determine what constitutes a public health crisis under the Doha Declaration. Wake Forest Intell. Prop. L.J. 10: 69-97

# Ghana

**Year(s):** 2005

**National Income:** Low

**Pharmaceutical Firm(s):** Various

**Drug(s):** ARVs

**Disease:** HIV/AIDS

**Disease Scenario Type:** HIV/AIDS

**Outcome(s):** CL

**Case Study Summary:** CPTech writes: “On October 26 2005, the Minister of Health issued a government use compulsory license for importation into Ghana of generic HIV-AIDS medicines.” Canadian-based efforts were made to supply the ARVS, but in the end, an Indian generic manufacturer was able to produce. Few details about this particular case study are available, and it was nearly excluded from the study for this reason; however, many academic and government papers list Ghana as one among those nations who has utilized compulsory licensing for pharmaceuticals. The actual compulsory license is posted on CPTech’s website. Future research will flush out more of these details.

**Selective Bibliography:**

Bucknell D (22 October 2007) The global IP scorecard for pharma and biotech compulsory licenses. Mondaq Business Briefing. Available: <http://www.mondaq.com/australia/article.asp?articleid=53442>. Accessed 5 June 2011.

CPTech (2010) Examples of health-related compulsory licenses. Available from <http://www.cptech.org/ip/health/cl/recent-examples.html>. Accessed: 5 June 2011.

Krohmal B (9 November 2006) Noah Novogrodsky on 'compulsory licensing in Ghana -the continuing barriers to affordable medicines. CPTech. Available: <http://www.cptech.org/blogs/drugdevelopment/2006/11/noah-novogrodsky-on-compulsory.html>. Accessed: 5 June 2011.

Panafrican News Agency (9 February 2006) Ghana announces arrival of more ARVs as patients picket. Available: LexisNexis.

Quashigah EK (26 October 2005) Notification of emergency and issuance of government use license. Ministry of Public Health, Ghana. Available: <http://www.cptech.org/ip/health/cl/Ghana.png>. Accessed: 5 June 2011.

# India

**Year(s):**  2006-2007

**National Income:** Lower Middle

**Pharmaceutical Firm(s):** Novartis

**Drug(s):** Imatinib Mesylate (Glivec or Gleevec)

**Disease:** Cancer (leukemia, stromal tumors)

**Disease Scenario Type:** NCD

**Outcome(s):** None

**Case Study Summary:**  Novartis obtained exclusive marketing rights (a precursor to pharmaceutical product patent protection) in 2003 and expected to obtain full product patent once the application was reviewed after the TRIPS compliance deadline in 2005. The Chennai Patent Office denied Novartis’ application on 25 January 2006, stating that the innovation was only a slight alternation of a known substance and lacked novelty. Novartis filed a lawsuit. Health Minister Anbumani Ramadoss demanded that Novartis withdraw its appeal and even warned that, “India hadn’t used compulsory licensing yet and ‘shouldn’t be pushed towards that” (quoted in David). David writes: “Generic versions of Glivec in India cost about one-tenth what Novartis charges for the drug every month, which is approximately 105,000 rupees ($2,600). That price is fixed globally, give or take variations from currency conversion. More than 6,700 people--about 99% of those who use Glivec in India--get treatment for free under a program Novartis runs with the Max Foundation.” In the end, the Madras High Court upheld the original product patent denial. While India has many cases of CL applications for export, e.g., to Nepal, the Financial Express confirms in 2010 that “India has never used compulsory licensing.”

**Selective Bibliography:**

David R (7 August 2007) Novartis set back in India patent fight over Glivec. Forbes. Available: <http://www.forbes.com/2007/08/07/novartis-glivec-generics-markets-equity-cx_rd_0807markets02.html>. Accessed 5 June 2011.

Financial Express (26 August 2010) Bad medicine. FE Editorial. Available: LexisNexis

Kaur M (22 November 2008) TRIPS and the Indian patent regime." SSRN. Available: <http://papers.ssrn.com/sol3/papers.cfm?abstract_id=1305585>. Accessed 5 June 2011.

Novartis Pharmaceuticals Corporation (2010) Glivec patent case in India: fact vs. fiction. Available: <http://www.novartis.com/downloads/about-novartis/facts-vs-fiction-india-glivec-patent-case.pdf>. Accessed 29 June 2010.

Raju K (20 November 2007) The debacle of Novartis patent case in India: strict interpretation of patentability criteria under Article 27 of the TRIPS Agreement. SSRN. Available: <http://papers.ssrn.com/sol3/papers.cfm?abstract_id=1030963>. Accessed 5 June 2011.

# Indonesia

**Year(s):** 2005

**National Income:** Lower Middle

**Pharmaceutical Firm(s):** Boehringer Ingelheim

**Drug(s):** Lamivudine (Epivir) and Nevirapine (Viramune)

**Disease:** HIV/AIDS

**Disease Scenario Type:** HIV/AIDS

**Outcome(s):** CL

**Case Study Summary:** Germano writes: “In January 2004, the President of Indonesia announced a plan to provide ARVs to approximately 5000 people living with HIV and AIDS over the course of the year. The government intended to increase the number of people treated by 50 percent in 2005. In 2004, the government issued 20 U.S. dollars per month per patient to subsidize the costs of the medicines; however, this effort was inadequate because the cost of a triple ARV regimen was almost 600 U.S. dollars each year per person.” The WHO reports: “On 5 October 2004, a presidential decree was issued in Indonesia authorizing the Minister of Health to appoint a manufacturer to exploit patents on lamivudine and nevirapine on behalf of the government. The decree specifies a royalty rate of 0.5% of the net (generic) sales price. The authorization lasts for 7 years (nevirapine) and eight years (lamivudine), i.e. for the remaining patent term.”

**Selective Bibliography:**

Bucknell D (22 October 2007) The global IP scorecard for pharma and biotech compulsory licenses. Mondaq Business Briefing. Available: <http://www.mondaq.com/australia/article.asp?articleid=53442>. Accessed 5 June 2011.

Germano S (2007) Compulsory licensing of pharmaceuticals in Southeast Asia: paving the way for greater use of the TRIPS flexibility in low- and middle-income countries. UMKC L Rev 76: 273-294.

Soekarnoputri M (6 December 2004) Text of Indonesia compulsory license. Available: <http://lists.essential.org/pipermail/ip-health/2004-December/007233.html>. Accessed 5 June 2011.

WHO (February 2008) Briefing note Access to medicines and countries experiences in using TRIPS safeguards. Available: <http://www.searo.who.int/LinkFiles/IPT_Briefing_note_4_country_experiences.pdf>Accessed 5 June 2011.

# Malaysia

**Year(s):** 2003-2004

**National Income:** Upper Middle

**Pharmaceutical Firm(s):** Bristol-Myers Squibb and GlaxoSmithKline

**Drug(s):** Didanosine (Videx), Zidovudine (Retrovir), and Lamivudine+Zidovudine

(Combivir)

**Disease:** HIV/AIDS

**Disease Scenario Type:** HIV/AIDS

**Outcome(s):** CL

**Case Study Summary:** The WHO writes: “On 29 October 2003…the authorization for the exploitation of a patented invention on behalf of the government...was issued. It allowed a local company to import didanosine tablets, zidovudine tablets and a fixed-dose combination (FDC) of didanosine+zidovudine from a generic manufacturer in India...The authorization was valid for two years. It required that the medicines be labelled with the words ‘Ministry of Health Malaysia’ and imposed several other conditions, including a maximum price and a requirement that royalties be paid to the patent holder(s). The MoH offered the patent holders 4% royalties. The patent holders however showed little interest in accepting or negotiating the proposed remuneration...Following the government use authorization, the patent holders reportedly reduced their prices by 50-80%.” Germano writes: “After the importation of the generic version of Didanosine, Zidovudine and Zidovudine + Lamivudine combination, the cost of treatment per month per patient dropped from 315 U.S. dollars to 58 U.S. dollars-an eighty-one percent decrease. In the short-term, the number of patients that could be treated in government facilities went from 1500 to 4000. The government hopes to reach its target of 10,000 after more outreach in the community.”

**Selective Bibliography:**

Germano S (2007) Compulsory licensing of pharmaceuticals in Southeast Asia: paving the way for greater use of the TRIPS flexibility in low- and middle-income countries. UMKC L Rev 76: 273-294.

Minister of Domestic Trade and Comer Affairs Malaysia (29 October 2003) Authorization for exploitation of patented invention in Malaysia. Available: <http://www.cptech.org/ip/health/c/malaysia/arv-license.html>. Accessed 5 June 2011.

WHO (February 2008) Briefing note Access to medicines and countries experiences in using TRIPS safeguards. Available: <http://www.searo.who.int/LinkFiles/IPT_Briefing_note_4_country_experiences.pdf>Accessed 5 June 2011.

# Mozambique

**Year(s):** 2004

**National Income:** LDC

**Pharmaceutical Firm(s):** GlaxoSmithKline, Bristol-Myers Squibb, Boehringer Ingelheim

**Drug(s):** Lamivudine (Epivir), Stavudine (Zerit), and Nevirapine (Viramune)

**Disease:** HIV/AIDS

**Disease Scenario Type:** HIV/AIDS

**Outcome(s):** CL

**Case Study Summary:** Dummett writes: “The Mozambican government has become the first in Africa to issue a compulsory license for the local manufacture of generic versions of patented HIV/AIDS drugs, in line with the World Trade Organization's Trade-Related Aspects of Intellectual Property Rights (TRIPS) agreement. The licence is understood to have been signed on 5 April [2004] this year, and was granted to domestic generics outfit Pharco Mozambique LDA. The company will manufacture fixed-dose combination therapies (comprising lamivudine, stavudine and nevirapine) under the brand Pharcovir 30 and Pharcovir 40. Pharco will pay royalties of 20% of revenue from the products. According to the government's licence, there were an estimated 100,000 patients with full-blown AIDS at end-2002, from an HIV+ population of 1.5m, or 8% of the total population. The government's licence justifies its decision on the grounds that the manufacturers of branded anti-retrovirals (GlaxoSmithKline, lamivudine; Bristol-Myers Squibb, stavudine; and Boehringer Ingelheim, nevirapine) failed to produce their own fixed-dose combination.”

**Selective Bibliography:**

Bucknell D (22 October 2007) The global IP scorecard for pharma and biotech compulsory licenses. Mondaq Business Briefing. Available: <http://www.mondaq.com/australia/article.asp?articleid=53442>. Accessed 5 June 2011.

CPTech (2010) Examples of health-related compulsory licenses. Available from <http://www.cptech.org/ip/health/cl/recent-examples.html>. Accessed 5 June 2011.

Dummett H (19 May 2004) Government issues HIV/AIDS compulsory license. World Markets Research Centre. Available from LexisNexis.

Namburete S (2004) COMPULSORY LICENCE no. 01/MIC/04. Available: <http://www.cptech.org/ip/health/c/mozambique/moz-cl-en.pdf>. Accessed 5 June 2011.

# Rwanda

**Year(s):** 2007

**National Income:** Low (least developed country status)

**Pharmaceutical Firm(s):** Apotex (Canadian generic supplier)

**Drug(s):** Lamivudine+Nevirapine+Zidovudine (FDC)

**Disease:** HIV/AIDS

**Disease Scenario Type:** HIV/AIDS

**Outcome(s):** CL

**Case Study Summary:** The WHO writes: “In July 2007, Rwanda notified the WTO secretariat of its intention to import 260 000 packs of a FDC of zidovudine+ lamivudine+nevirapine from Apotex, a generic manufacturer in Canada. This is the first attempt to make use of this system. The notification states that Rwanda reserves the right to modify the quantity as necessary. It furthermore states that Rwanda will make use of its right, as a least-developed country, not to enforce any patent rights that may have been granted with regard to this product. Following this request, the Canadian Commissioner of Patents granted, in September 2007, a CL to Apotex, allowing Apotex to manufacture the concerned product exclusively for export to Rwanda. This CL is valid for a period of two years.”

**Selective Bibliography:**

Africa News (28 April 2008) Rwanda, Africa's failing infrastructure renders compulsory licensing pointless. Available from LexisNexis.

Feldman J (2009) Compulsory licenses: the dangers behind the current practice. J Int’l Bus & L 8: 137-167.

Tsai G (2009) Canada’s access to medicines regime: lessons for compulsory licensing schemes under the WTO Doha Declaration. Va J Int'l L 49:1063-1097.

WHO (February 2008) Briefing note Access to medicines and countries experiences in using TRIPS safeguards. Available: <http://www.searo.who.int/LinkFiles/IPT_Briefing_note_4_country_experiences.pdf>Accessed 5 June 2011.

WTO (2010) TRIPS and public health paragraph 6 system: Notifications by importing WTO members. Available: <http://www.wto.org/english/tratop_e/trips_e/public_health_notif_import_e.htm>. Accessed 5 June 2011.

# South Africa

**Year(s):** 2001-2003

**National Income:** Lower Middle

**Pharmaceutical Firm(s):** Various

**Drug(s):** Nevirapine (Viramune), Lamivudine (Epivir), Zidovudine (Retrovir), Stavudine (Zerit), Didanosine (Videx), Efavirenz (Stocrin or Sustiva), Indinavir (Crixivan), and Abacavir (Ziagen)

**Disease:** HIV/AIDS

**Disease Scenario Type:** HIV/AIDS

**Outcome(s):** Discount/VL/None

**Case Study Summary:** This case study includes a web of splintering stories, all of which have a very similar starting point—the “Medicines and Related Substances Control Amendment Act, No. 90 of 1997. This Act was designed to increase citizens’ access to otherwise unaffordable drugs. In 1998, the South African Pharmaceutical Manufactures Associates along with 40 multinational pharmaceutical giants filed a lawsuit, arguing the legislation contradicted both TRIPS and the South African Constitution. CPTech writes, “on March 7, 2001, Indian pharmaceutical manufacturer CIPLA formally requested the South African Department of Trade and Industry to issue compulsory licenses to patents on the following HIV drugs: nevirapine, lamivudine, zidovudine, stavudine, didanosine, efavirenz, indinavir and abacavir.” The case received significant international media attention as the US and the European Commission applied pressure upon South Africa to rescind the legislation. The case reached the courtroom in May 2000; but the firms dropped the lawsuit in April 2001 after withstanding substantial criticism in the international media.

**Selective Bibliography:**

Block R (12 March 2001) Cipla sidesteps South African fight with a bid to offer generic drugs. Wall Street Journal. Available at <http://www.aegis.org/news/wsj/2001/WJ010309.html>. Accessed 5 June 2011.

Hoen ET (2002) Public health and international law: TRIPS, pharmaceutical patents, and access to essential medicines: a long way from Seattle to Doha. Chi J Int’l L 3: 27-46.

Swarns R (8 March 2001) AIDS drug battle deepens in Africa. New York Times. Available: <http://www.nytimes.com/2001/03/08/world/aids-drug-battle-deepens-in-africa.html?src=pm>. Accessed 5 June 2011.

WHO (February 2008) Briefing note Access to medicines and countries experiences in using TRIPS safeguards. Available: <http://www.searo.who.int/LinkFiles/IPT_Briefing_note_4_country_experiences.pdf>Accessed 5 June 2011.

# Taiwan

**Year(s):** 2005

**National Income:** High

**Pharmaceutical Firm(s):** Roche

**Drug(s):** Osetamivir (Tamiflu)

**Disease:** Avian flu/Pandemic Flu

**Disease Scenario Type:** CD

**Outcome(s):** VL

**Case Study Summary:** International Centre for Trade and Sustainable Development reports: “Taiwan has become the first country to issue a compulsory licence for the generic production of Tamiflu, the only drug currently available for the treatment of avian flu, in order to ensure that the country has sufficient quantities of the medicine in the event of a pandemic.” Leonard writes: “Taiwan considers itself on the front lines of a potential bird flu pandemic...Unsure that Roche would be able to live up to its promise to provide enough Tamiflu to cover a sufficient percentage of Taiwan's population, Taiwan decided to plunge ahead. In November 2005, Taiwan's government issued a license to allow local companies to manufacture generic versions of Tamiflu -- the only drug in the world considered effective in combatting the effects of bird flu...[Taiwan amended] its patent laws to allow the export of its generics to other nations.” Roche confirms that it “will provide Taiwan with an additional 1.3 million treatments of Tamiflu (oseltamivir) next year, taking the total treatments of Tamiflu ordered for pandemic use in the country to 2.3 million treatments. This will be used to safeguard the public health of citizens in Taiwan in the event of an influenza pandemic. Roche’s ability to meet the needs of Taiwan has been made possible due to a global scale-up effort that has been underway at Roche since 2004, resulting in a production output by the end of 2006 of 300 million treatments a year.”

**Selective Bibliography:**

International Centre for Trade and Sustainable Development (30 November 2005) Taiwan issues compulsory license for Tamiflu. Available: <http://ictsd.org/i/ip/39838/>. Accessed: 11 June 2011.

Leonard A (6 September 2006) Adventures with Tamiflu: is Taiwan throwing down the big gauntlet at big pharma? Salon. Available: <http://www.salon.com/technology/how_the_world_works/2006/09/06/taiwan_tamiflu>. Accessed 5 June 2011.

Nag AD (2006-2007) The bird flu and the invoking of TRIPS Article 31 "National Emergency" Exception. Syracuse J Int’l L & Com 34: 689-713.

Roche (25 November 2005) Media release: Roche confirms ability to supply pandemic Tamiflu to the people of Taiwan. Available: <http://www.roche.com/med-cor-2005-11-25>. Accessed 5 June 2011.

# Thailand (and Merck)

**Year(s):** 2006 (2010)

**National Income:** Lower Middle

**Pharmaceutical Firm(s):** Merck

**Drug(s):** Efavirenz (Stocrin or Sustiva)

**Disease:** HIV/AIDS

**Disease Scenario Type:** HIV/AIDS

**Outcome(s):** CL

**Case Study Summary:** The WHO writes: “Thailand issued its first CL in November 2006, for efavirenz. About two months later, the first consignment of generic efavirenz was imported from India, at half the original price...Generic efavirenz [was] being imported...initially. Meanwhile national companies have started preparations for local production.” Shashikant notes that: “the cost of the drug for treatment will be USD 22 per month using the Indian generic version compared to the Merck price in which is USD 41 per month…Payment equivalent to 0.5 per cent of the sales in Thailand for the generic version of efavirenz will be made to Merck.” This CL was renewed in 2010.

**Selective Bibliography:**

Bangkok Post (16 June 2010) The licensing of key drugs. Available: <http://www.bangkokpost.com/opinion/opinion/38827/the-licensing-of-key-drugs>. Accessed 5 June 2011.

Fuller T (11 April 2007) Thailand takes on drug industry, and may be winning. New York Times. Available: <http://www.nytimes.com/2007/04/11/world/asia/11iht-pharma.4.5240049.html?_r=1&pagewanted=1>. Accessed 5 June 2011.

Merck (2009-2010) Improving access to HIV and AIDS treatment and care. Available: <http://www.merck.com/corporate-responsibility/access/access-hiv-aids/approach.html>. Accessed 5 June 2011.

Shashikant S (6 December 2006) TWN Info Service on WTO and Trade Issues. Third

World Network. Available: <http://www.twnside.org.sg/title2/twninfo486.htm>. Accessed 5 June 2011.

WHO (February 2008) Briefing note Access to medicines and countries experiences

in using TRIPS safeguards. Available: <http://www.searo.who.int/LinkFiles/IPT_Briefing_note_4_country_experiences.pdf>Accessed 5 June 2011.

# Thailand (and Abbott, Sanofi-Aventis)

**Year(s):** 2007 (2010)

**National Income:** Lower Middle

**Pharmaceutical Firm(s):** Abbott and Sanofi-Aventis

**Drug(s):** Lopinavir+ritonavir (Kaletra) and Clopidogrel (Plavix)

**Disease:** HIV/AIDS and cardiovascular disease

**Disease Scenario Type:** HIV/AIDS and NCD

**Outcome(s):** CL

**Case Study Summary:** The WHO writes: “In January 2007, two more CLs were issued, for lopinavir/ritonavir [Kaltera] and for a cardiovascular drug, clopidogrel [Plavix]. This was the first time a developing country used compulsory licensing in relation to a non-communicable disease… [and it] generated controversy.” Fuller writes: “Abbott…cut the price of Kaletra in low- and medium-income countries, including Thailand, to $1,000 a patient per year. That is less costly than any generic on the market and 55 percent less than the current price, the company said.” The Kaletra CL was renewed in June 2010, according to the Bangkok Post. The Third World Network reports that clopidogrel was produced by Indian firm M-Cure and that the cost was only 70 baht per tablet (the market cost was 1.01 baht per tablet).

**Selective Bibliography:**

Fuller T (11 April 2007) Thailand takes on drug industry, and may be winning. New York Times. Available: <http://www.nytimes.com/2007/04/11/world/asia/11iht-pharma.4.5240049.html>. Accessed 5 June 2011.

Ministry of Public Health and The National Health Security Office of Thailand (February 2007) Facts and evidences on the 10 burning issues related to the government use of patents on three patented essential drugs in Thailand. Available: <http://www.moph.go.th/hot/White%20Paper%20CL-EN.pdf>. Accessed 5 June 2011.

Savoie B (2007-2008) Thailand's test: compulsory licensing in an era of the epidemiological transition. Va J Int’l L 48: 211-248.

Third World Network (29 August 2007) TWN Info Service on Trade and WTO Issues. Available: <http://www.twnside.org.sg/title2/wto.info/twninfo080706.htm>. Accessed 5 June 2011.

WHO (February 2008) Briefing note Access to medicines and countries experiences in using TRIPS safeguards. Available: <http://www.searo.who.int/LinkFiles/IPT_Briefing_note_4_country_experiences.pdf>Accessed 5 June 2011.

# Thailand (and Novartis)

**Year(s):** 2007-2008

**National Income:** Lower Middle

**Pharmaceutical Firm(s):** Novartis

**Drug(s):** Imatinib Mesylate (Glivec or Gleevec)

**Disease:** Cancer (leukemia, stromal tumors)

**Disease Scenario Type:** NCD

**Outcome(s):** Discount

**Case Study Summary:** Fuller writes: “The Swiss drug company Novartis offered an effective 75 percent price reduction this week in its leukemia medicine, Glivec, after Thai officials said they were studying a compulsory license on the drug, which would have allowed the government to produce it in its own factories and distribute it on a nonprofit basis.” The Agence French Presse adds: “This CL has been considered intermittently throughout the Thailand saga; however, Novartis seemed to prevent the CL by always offering greater discounts. Later in 2007, Novartis arranged to provide the drug to Thailand for free.”

**Selective Bibliography:**

Agence French Presse (21 November 2007) Thailand may get cancer drug free from Novartis. Available: LexisNexis.

Fuller T (11 April 2007) Thailand takes on drug industry, and may be winning. New York Times. Available: <http://www.nytimes.com/2007/04/11/world/asia/11iht-pharma.4.5240049.html?_r=1&pagewanted=1>. Accessed 5 June 2011.

Pharma Marketletter (13 March 2008) New Thailand govt's compulsory drug licensing policy swings back and forth. Available: LexisNexis.

Thai Press Reports (25 September 2007) Thailand compulsory licensing to expand to cancer drugs. Available: LexisNexis.

# Thailand (and Novartis, Sanofi-Aventis, Roche)

**Year(s):** 2007-2008

**National Income:** Lower Middle

**Pharmaceutical Firm(s):**  Novartis, Sanofi-Aventis, Roche

**Drug(s):**  Letrozole (Femara), Docetaxel (Taxotere), and Erlotinib (Tarceva)

**Disease:** Cancers (Femara is used primarily to treat breast cancer; Taxotere and Tarceva are used primarily for lung cancer)

**Disease Scenario Type:** NCD

**Outcome(s):** CLs

**Case Study Summary:**  The Thai Minster of Health, Mongkol na Songkhla initiated the second wave of CLs later in 2007. The CLs were for Novartis’ Letrozole, Sanofi-Aventis’ Docetaxel, and Roche Erlotinib, which are used to treat varying types of cancers including those of the breast, lung, ovaries, and pancreas. The Third World Network notes that “according to the former Minister [of Health,] there are about 15,000 people in Thailand with lung and liver cancer who are in need of the drugs.” Mongkol na Songkhla was replaced in early 2008, however, by Chaiya Sasomsab, who threatened to rescind this second batch of CLs shortly after taking his post as the new Minister of Health. After sustaining substantial criticism from the general Thai public, international NGOs, and patient groups, Chaiya Sasomsab announced that there were major legal obstacles and that he was unable to revoke the CLs.

**Selective Bibliography**:

Pharma Marketletter (13 March 2008) New Thailand govt's compulsory drug licensing policy swings back and forth. Available: LexisNexis.

Sajirawattanakul D (23 February 2008) Drug-licensing revision should be ready by April. Available: <http://unitcost.fda.moph.go.th/cl/news_detail.php?id=868&type=5>. Accessed 5 June 2011.

The Nation – Thailand (8 March 2008) Generic drug debate intensifies. Available: <http://unitcost.fda.moph.go.th/cl/newsfile_file/newsfile_20080910044737-1.pdf>. Accessed 5 June 2011.

Thai Press Reports (25 September 2007) Thailand compulsory licensing to expand cancer drugs. Available: LexisNexis.

Third World Network (25 February 2008) TWN Info Service on Health Issues. Available: <http://www.twnside.org.sg/title2/health.info/2008/twnhealthinfo20080201.htm>. Accessed 5 June 2011.

# United States

**Year(s):** 2001

**National Income:** High

**Pharmaceutical Firm(s):** Bayer

**Drug(s):** Ciproflaxin (Cipro)

**Disease:** Anthrax

**Disease Scenario Type:** CD

**Outcome(s):** Discount

**Case Study Summary:**  The United States examined its CL policy after bioterrorist-engineered anthrax spores were mailed to political and news entities around the country. The nation began to fear a shortage of Cipro and the Secretary of the Health and Human Services proceeded to negotiate with the patent titleholder. A substantial discount was agreed upon. Bradsher writes: “Bayer agreed to ship 100 million tablets of Cipro by the end of the year for 95 cents each, and to donate another 2 million tablets beginning next week. The government also obtained options to buy an additional 100 million tablets after this year at 85 cents each, and a further 100 million tablets for 75 cents a piece.”

**Selective Bibliography:**

Bayer Corporation (24 June 2002) Form 20-F. United States Securities and Exchange Commission. Available: [www.investor.bayer.com/user_upload/469/](http://www.investor.bayer.com/user_upload/469/). Accessed 5 June 2011.

Bradsher K (25 October 2001) Bayer agrees to charge government a lower price for anthrax medicine. New York Times. Available: <http://www.nytimes.com/2001/10/25/business/25BAYE.html>. Accessed on 5 June 2011.

Curti AM (2001) The WTO dispute settlement understanding: an unlikely weapon in the Fight against AIDS. AM JL & Med 27: 469-485.

Ferrone JD (2003) Compulsory licensing during public health crises: bioterriorisms mark on global pharmaceutical patent protection. Suffolk Transnatl Law R 26: 385-410.

Murthy D (2002) The future of compulsory licensing: deciphering the Doha declaration on the TRIPs agreement and public health. Am U Int’l L Rev 17: 1299-1346.

# Zambia

**Year(s):** 2004

**National Income:** LDC

**Pharmaceutical Firm(s):** Boehringer-Ingelheim and Bristol-Myers Squibb

**Drug(s):** Lamivudine (Epivir), Stavudine (Zerit), and Nevirapine (Viramune)

**Disease:** HIV/AIDS

**Disease Scenario Type:** HIV/AIDS

**Outcome(s):** CL

**Case Study Summary:** The WHO writes: “On 29 September 2004, Zambia issued a CL to allow a domestic company to manufacture a FDC of lamivudine, stavudine, and nevirapine. The CL prohibits export, and specifies that the total amount of royalties payable to the patent holder(s) shall not exceed 2.5% of the turnover of the product.”

**Selective Bibliography:**

Chilrpamushi DM (30 September 2004) Notice of issuance of compulsory license. Available: <http://www.cptech.org/ip/health/c/zambia/zambia-bms09302004.html>. Accessed 5 June 2011.

Patel DK (22 Sep 2004) Compulsory License No. CL 01/2004. Available: <http://www.cptech.org/ip/health/c/zambia/zcl.html>. Accessed 5 June 2011.

Times of Zambia (23 September 2004) Company granted license to make ARVs locally. Available: LexisNexis.

WHO (February 2008) Briefing note Access to medicines and countries experiences in using TRIPS safeguards. Available: <http://www.searo.who.int/LinkFiles/IPT_Briefing_note_4_country_experiences.pdf>Accessed 5 June 2011.

# Zimbabwe

**Year(s):** 2003

**National Income:** Low

**Pharmaceutical Firm(s):** Varichem Pharmaceuticals Ltd (local generic manufacturer)

**Drug(s):** All ARVs

**Disease:** HIV/AIDS

**Disease Scenario Type:** HIV/AIDS

**Outcome(s):** CL

**Case Study Summary:** The WHO writes: “On 8 April 2003, Zimbabwe issued a CL for all HIV and AIDS- related medicines. The license was issued after a period of emergency on HIV/AIDS was declared. The CL allows a local company, Varichem Pharmaceuticals Ltd, to produce ARVs or HIV/AIDS-related medicines during the emergency period. The license requires the company to supply three quarters of its production to state-owned health institutions and specifies that the medicines produced under the license will be subject to price controls. Varichem reportedly launched its first ARV in Zimbabwe in October 2003, and has since launched several other ARVs. It supplies to both the government and private sector.” Bucknell writes: “In 2003, the period of emergency was extended by five years [expiring in] until 31 December 2008.”

**Selective Bibliography:**

Bucknell D (22 October 2007) The global IP scorecard for pharma and biotech compulsory licenses. Mondaq Business Briefing. Available: <http://www.mondaq.com/australia/article.asp?articleid=53442>. Accessed 5 June 2011.

CPTech (2010) Examples of health-related compulsory licenses. Available from <http://www.cptech.org/ip/health/cl/recent-examples.html>. Accessed 5 June 2011.

Rajesh D (31 March 2010) The efficacy of compulsory licensing of pharmaceutical patents. Mondaq. Available: LexisNexis.

WHO (February 2008) Briefing note Access to medicines and countries experiences in using TRIPS safeguards. Available: <http://www.searo.who.int/LinkFiles/IPT_Briefing_note_4_country_experiences.pdf>Accessed 5 June 2011.
